# Geranyl Functionalized Materials for Site-Specific Co-Immobilization of Proteins

**DOI:** 10.3390/molecules26103028

**Published:** 2021-05-19

**Authors:** Jana Brabcova, Alicia Andreu, David Aguilera, Zaida Cabrera, Blanca de las Rivas, Rosario Muñoz, Jose M. Palomo

**Affiliations:** 1 Department of Biocatalysis, Institute of Catalysis (CSIC), Marie Curie 2, 28049 Madrid, Spain; brabcova@uochb.cas.cz (J.B.); aliandreuvillas@gmail.com (A.A.); aguilera.rgz@gmail.com (D.A.); 2 Bacterial Biotechnology, Institute of Food Science, Technology and Nutrition (ICTAN-CSIC), Juan de la Cierva 3, 28006 Madrid, Spain; blanca.r@csic.es (B.d.l.R.); r.munoz@csic.es (R.M.); 3 School of Biochemical Engineering, Pontificia Universidad Católica de Valparaíso, Avda, Brasil, 2085 Valparaíso, Chile; zaida.cabrera@pucv.cl

**Keywords:** geranyl, functionalized materials, hydrophobic interactions, thiol-alkene reaction, seleno-alkene reaction, azide cycloaddition, click chemistry, enzymes

## Abstract

Different materials containing carboxylic groups have been functionalized with geranyl-amine molecules by using an EDC/NHS strategy. Chemical modification of the support was confirmed by XRD, UV-spectrophotometer, and FT-IR. This geranyl-functionalized material was successfully applied for four different strategies of site-selective immobilization of proteins at room temperature and aqueous media. A reversible hydrophobic immobilization of proteins (lipases, phosphoglucosidases, or tyrosinase) was performed in neutral pH in yields from 40 to >99%. An increase of the activity in the case of lipases was observed from a range of 2 to 4 times with respect to the initial activity in solution. When chemically or genetically functionalized cysteine enzymes were used, the covalent immobilization, via a selective thiol-alkene reaction, was observed in the presence of geranyl support at pH 8 in lipases in the presence of detergent (to avoid the previous hydrophobic interactions). Covalent attachment was confirmed with no release of protein after immobilization by incubation with hydrophobic molecules. In the case of a selenium-containing enzyme produced by the selenomethionine pathway, the selective immobilization was successfully yielded at acidic pH (pH 5) (89%) much better than at pH 8. In addition, when an azido-enzyme was produced by the azide–homoalanine pathway, the selective immobilization was successful at pH 6 and in the presence of CuI for the click chemistry reaction.

## 1. Introduction

The immobilization of biomolecules represents an important alternative for their technological applications in different areas such as pharmaceuticals, textile, environmental, etc. [1,2,3,4,5]. The development of immobilization strategies of enzymes has been greatly increased in the last 20 years in order to provide economical and robust biocatalysts for the industry. One important aspect is the recovery of the biocatalyst, but also, the improvement of enzyme stability, activity, and selectivity are key issues in the development of new immobilization strategies [6,7,8]. In particular, in lipases, one of the most versatile types of enzymes used in biotransformations, immobilization techniques have also been applied to improve the activity or enantio and regioselectivity of the enzymes [9,10,11,12].

Currently, the economization in the final production or the extreme selectivity of the conjugation of an enzyme to a solid support are of relevant interest. The first means the development of a strategy to achieve the synthesis of a particular product from a starting material through a cascade process, which involves the use of two or more enzymes [13,14,15,16,17] or the combination of enzymes, artificial metalloenzymes, or even hybrid systems with transition metal catalysts [18,19,20,21]. This process will perform multiple catalytic steps in a single container, so it greatly diminishing disadvantages such as purification stages, synthetic steps, high amount of wastes, and the drastic reduction of the final performance of the process. For this, the development of a system that allows the immobilization of different enzymes on the same functionalized support represents a challenge [22]. Most of the reported methods focus on the manufacture of materials that present orthogonal functional reactive groups to immobilize proteins in different ways [23].

To achieve this, the synthesis of functionalized materials with a unique versatile chemical group [24,25] for this purpose is necessary. In this term, the prenyl group (diene group) meets this requirement. Diene can react through reversible interactions via π-alkene and alkane-alkene interactions and by a selective irreversible covalent mechanism with various nucleophiles such as SH, Se, or N_3_ through different types of chemistries [26,27,28,29] in the presence of free amino groups.

**Figure 1:**
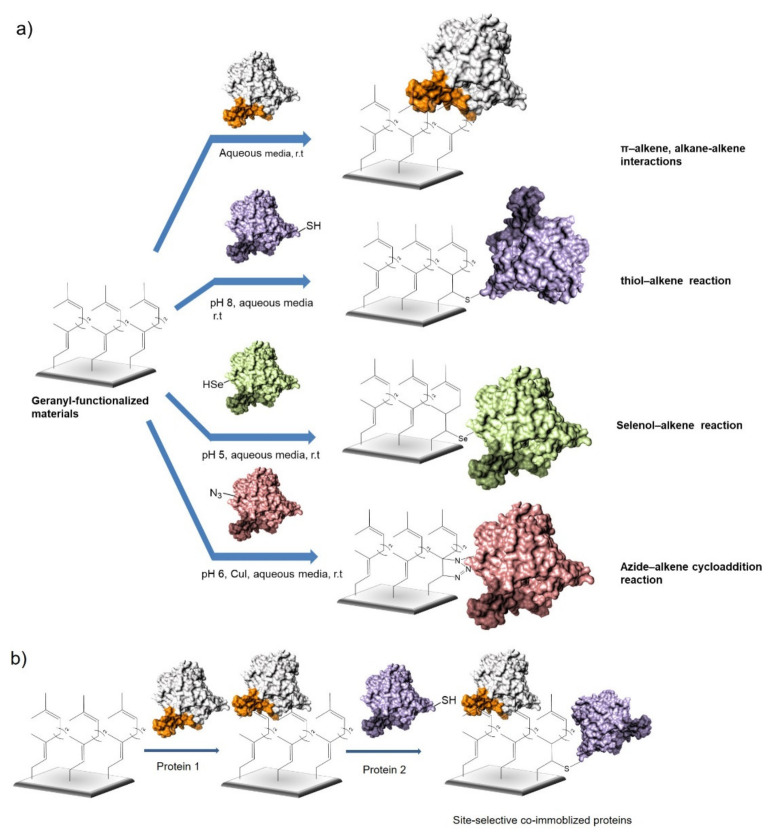
Site-selective immobilization of proteins on geranyl-functionalized materials. (**a**) Potential different immobilization strategies on geranyl supports. (**b**) Technology for site-selective co-immobilization of two different proteins under mild conditions.

Here, we present for the first time the application of this prenyl group, in particular the geranyl group, in a functionalized material for the specific immobilization of enzymes by different ways (Figure 1a), being a new alternative for simple enzymes co-immobilization (Figure 1b).

Lipases were successfully immobilized by different strategies, and it was possible to combine more than one at the same time with different chemistry, from a simple hydrophobic adsorption to covalent attachment at pH 8 (SH), 5 (Se), or 6 (N_3_). The versatility and capacity of this functionalized support was also studied with other enzymes. The general scheme of the tailor-made immobilization of enzymes on geranyl support is described in Figure 1. This new strategy could be a fine alternative to develop novel kinds of biosensors or applications in chemical cascade processes for the more sustainable production of drug intermediates.

## 2. Results and Discussion

### 2.1. Synthesis of Geranyl-Functionalized Materials

**Figure 2:**
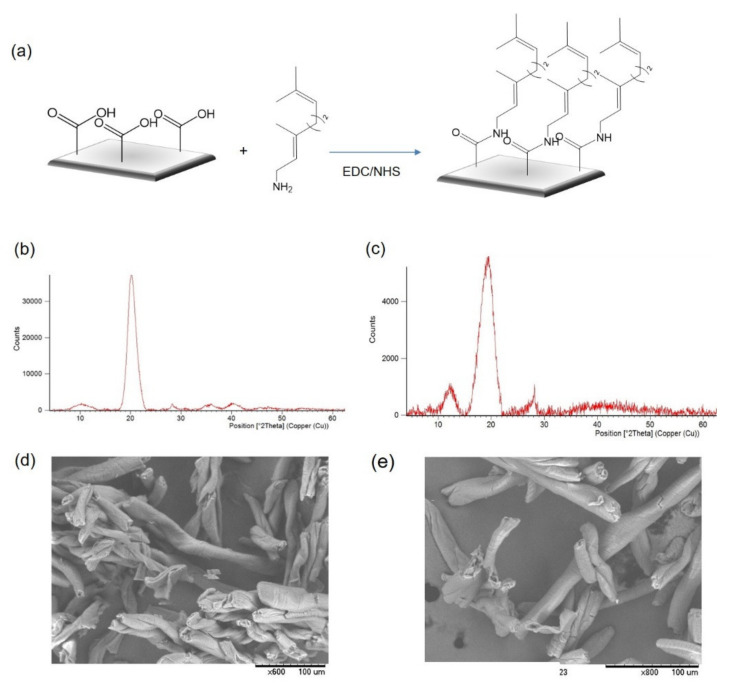
Geranyl functionalized materials synthesis. (**a**) Scheme of the process. (**b**) XRD patterns of CM. (**c**) XRD pattern of CM–geranyl support. (**d**) SEM image of CM. (**e**) SEM images of CM–geranyl.

Different commercial available materials containing carboxylic groups on the surface, iminodiacetic acid (IDA)-Sepabeads, and carboxymethylcellulose (CM) were successfully modified with geranyl groups via a typical amidation reaction by the EDC/NHS approach [12]. Geranyl-amine was incorporated on the support by a previous carboxylic groups activation with 1-ethyl-3-(3-dimethylaminopropyl) carbodiimide (EDC) in water at pH 4.5 and then activation with *N*-hydroxysuccinamide (NHS) at pH 7. Activated support was incubated with geranyl-amine overnight (Figure 2a).

The incorporation of the geranyl groups to the support was confirmed by X-ray diffraction (XRD) (Figure 2b,c).

The XRD pattern in the modified CM clearly suffered a decrease and, more importantly, a shift in the main peak around 20° and another at 10° degrees, whereas the peaks corresponding to the COO disappeared, indicating the formation of the amide group and therefore the incorporation of the geranyl group in the matrix. Scanning electron microscope (SEM) analysis of the CM support before and after the modification also demonstrated that no changes in the material morphology was produced with the chemical introduction of the geranyl groups (Figure 2d,e, Figure S1 and Figure S2). A similar result was observed in the Sepabeads (SP) material (Figure S3).

### 2.2. Selective Immobilization of Enzymes on Geranyl Support by Hydrophobic Interactions

First, the novel geranyl support was applied for direct immobilization of enzymes at neutral pH (Table 1). Considering the nature of the support, a reversible immobilization through hydrophobic interactions was expected.

Considering the capacity of lipases (acyl hydrolases) to be selectively absorbed on hydrophobic materials, fixing the open conformation of them [30,31], they were first tested.

At neutral pH and room temperature, different lipases in protein ranging from 3.4 to 12 mg were successfully immobilized with yields between 70 and >95% (Table 1, entries 1–7).

Interestingly, this methodology allowed the hyperactivation of *Candida antarctica* B lipase (CAL-B) after coordination with the support. If compared with the immobilization of the same enzyme on octyl-Sepharose (Table S1), the retained activity of enzyme, comparing with the initial soluble enzyme as 100%, in this immobilized form was 80%.

A similar case was observed also in *Rhizomucor miehei* lipase (RML), where the enzyme immobilized on the geranyl support showed almost two times more activity compared with the soluble one, whereas the activity hardly changed when immobilizing on octyl-Sepharose support (Table S1).

However, the effect was the opposite for other lipases such as *Candida rugosa* lipase (CRL), *Pseudomonas fluorescens* lipase (PFL), or *Aspergillus niger* lipase (ANL) (Table 1, entries 4–6, Table S1).

A possible explanation could be based on the structural differences between the lipases; for example CALB or RML present a shorter and different oligopeptide lid [32].

Furthermore, this selective methodology of immobilization of lipases allowed the direct purification of them, for example in the case of CALB, RML, or *Thermomyces* *lanuginosus* lipase (TLL) (Figure S4).

This immobilization technology was also successful for other kinds of enzymes, such as tyrosinase from mushroom, obtaining 40% yield in geranyl-CM and 90% in geranyl-SP (Table 1, entries 8-9), or even two novel phosphoglucosidases from *L. plantarum* WCFS1, Lp_0440 and Lp_3525, where in one case, >95% immobilization yield was observed (Table 1). These two proteins have a particularity because they presented a (His)_6_-tag in the *C*- or *N*-terminus, respectively (Figure S5). This could be important for the possible immobilization on this support, maybe by alkene–alkene type interactions, although the influence of the global hydrophobicity of the surface must be considered. This could be possible in terms of these kinds of interactions more than other types upon comparing the results with β-galactosidases (Table 1), which are not immobilized on geranyl support, but they were in octyl-Sepharose (Table S1).

Furthermore, in order to evaluate the co-immobilization capacity, CAL-B and tyrosinase were sequentially immobilized in geranyl-SP support, and the supported enzymes showed activity toward pNPB and l-DOPA.

### 2.3. Selective Covalent Immobilization of Enzymes on Geranyl Support by Thiol-Alkene Reaction

The second approach was the covalent selective immobilization of SH-containing proteins by a thiol-alkene modification between the SH in the protein and the double bond of the geranyl group.

**Figure 3:**
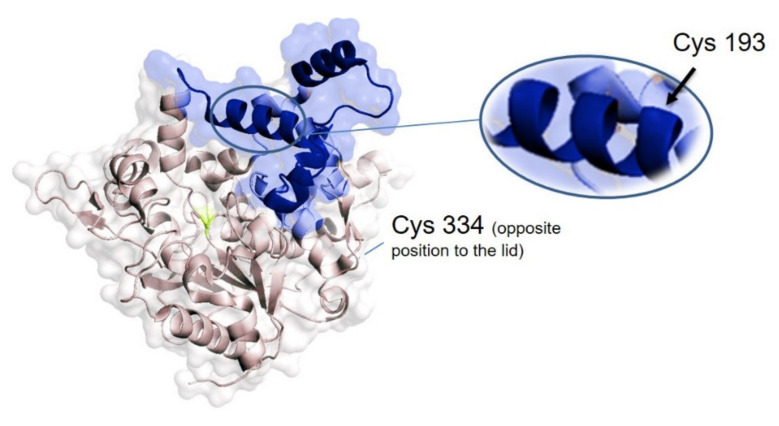
Three-dimensional structure of the *G. thermocatenulatus* lipase (GTL) marking the position of the different variants. The protein structure was obtained from the Protein Data Bank (pdb code: 2W22) and the picture was created using Pymol v. 0.99.

In order to perform that, genetically and chemically enzymes with SH were prepared. In the former case, thermophilic lipase from *Geobacillus* *thermocatenulatus* (GTL) (formerly called *Bacillus thermocatenulatus* lipase BTL2) was genetically engineered to produce two different variants containing a unique cysteine in a specific position, GTLC193, with the cysteine in the oligopeptide lid, and GTLC334, with the cysteine located in the opposite site of the active site (Figure 3) [33]. 

It has been recently demonstrated that the chemical modification or immobilization of these two enzyme variants are key in the selectivity of the enzyme [34,35].

First, the GTLC193 was incubated together with geranyl support at different pHs (from 5 to 8). In all cases, the addition of 0.1%TritonX-100 was performed to avoid any other immobilization mechanism (by hydrophobic interaction).

As described in Table 2, GTLC193 was immobilized on geranyl support by the selective covalent thiol-alkene reaction, being clearly more effective at alkaline pH, where 42% yield was observed after 2 h of incubation, with a retained activity higher than 80%.

Using the optimal conditions, the GTL variant (GTLC334) was also immobilized by this method with an excellent yield (68%), in this case retaining around 50% of its initial activity (Table 2).

**Figure 4:**
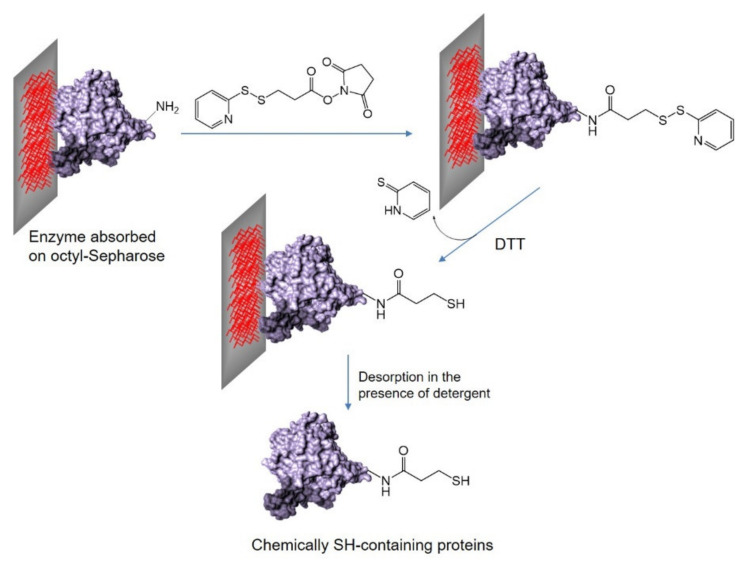
Scheme of the strategy for the chemical introduction of SH groups on the protein surface. (NH_2_ is referred to the *N*-terminal amino). DTT: dithiothreitol.

In order to extend the strategy, a chemical incorporation of the SH group to the protein surface was attempted (Figure 4). For that, succinimidyl 3-(2-pyridyldithio)propionate (SPDP) was used to perform a direct modification of the N-terminus of the protein. For this study, two different enzymes were modified, Rhizomucor miehei lipase (RML) and β-galactosidase from E. coli (β-Gal). This modification process was performed on solid phase; both enzymes were previously absorbed on octyl-Sepharose (Table S1). Then, the solution of SPDP was added at pH 7.3. In order to obtain a free SH on the protein, a DTT treatment was used (Figure 4). Finally, the chemical modified RML_SH and B-Gal_SH were recovered in solution by adding a detergent.

These modified enzymes, which already contained the detergent (avoiding undesired interactions), were incubated in the presence of geranyl support at pH 8. Very good immobilization yields were obtained, in particular for B-Gal (79% yield) conserving the complete initial activity in solution on the solid.

In order to confirm that the immobilization proceeded by a covalent interaction and was not ionic, after removing the detergent, the solid—in both cases—was incubated in a solution of NaCl (1 M) with no protein release from the solid (data not shown). In addition, the incubation of the solid with 3% (v/v) of detergent was tested, and again, no protein release in the supernatant was observed (data not shown).

### 2.4. Selective Covalent Immobilization of Seleno Enzymes on Geranyl Support by Selenol-Alkene Reaction

The incorporation of selenol (SeH) to the enzymes has been well described by an auxotrophic expression, and the main advantage of the introduction of this functional group is its unique nucleophilic properties (pKa 5.2) that allow it to be selectively modified even in the presence of cysteine (pKa 8). This is important if the protein is sensitive to neutral-alkaline pH.

In order to demonstrate that, a seleno-protein was developed directly on the variant GTLC193 (which contains a unique cysteine). This was performed by incubating *E. coli* BL21 (DE3) containing the plasmid pT1GTLmutCys-A193C in the presence of selenomethionine. The GTL enzyme showed seven different methionine residues in the structure with three of them accessible on the surface (ESI, Figure S6 and Figure S7), which can be modified.

Then, the GTLC193_SeH was purified by a hydrophobic chromatography sequence (Figure S8).

**Figure 5:**
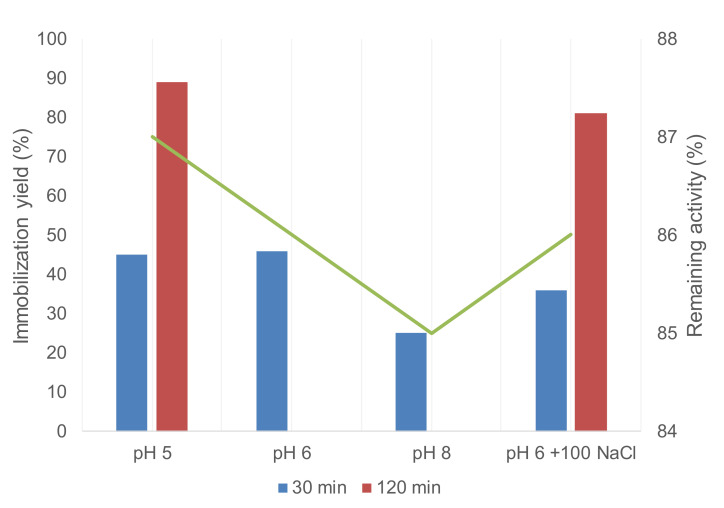
Immobilization of GTLC193_SeH on geranyl-SP support at different conditions. Remaining activity (green).

Therefore, similar to GTLC193, the GTLC193_SeH variant was immobilized on geranyl support at different pHs (Figure 5).

The immobilization yield was higher at lower pH; it was much better at pH 5, where around 90% yield was achieved after 2 h incubation (Figure 5). More than 80% activity was conserved on the solid in all cases. In addition, the effect of the presence of more ionic strength in the media was tested. In this case, no significant variation in the immobilization process was observed, demonstrating that the protein immobilization was by a selenol-alkene reaction (Figure 1).

Just to confirm that, the immobilized derivative was incubated in 500 mM NaCl and then washed with distilled water, and no loss of activity on the solid was observed (data not shown).

### 2.5. Selective Covalent Immobilization of Azido Enzymes on Geranyl Support by Azide-Cycloaddition Reaction

Another orthogonal chemical strategy for protein modification is the click chemistry [36] and in particular the 1,3 dipolar azide cycloaddition reaction [37,38].

In this term, this reaction proceeds in a selective and efficient way at almost neutral pH in the presence of cupric iodide (CuI) at room temperature [36,38].

Following the procedure described in Section 2.4, a new variant of GTLC193 was created by using azidehomoalanine as an exchanger amino acid, obtaining the new azido protein GTLC193_N_3_ (Figure S8).

**Figure 6:**
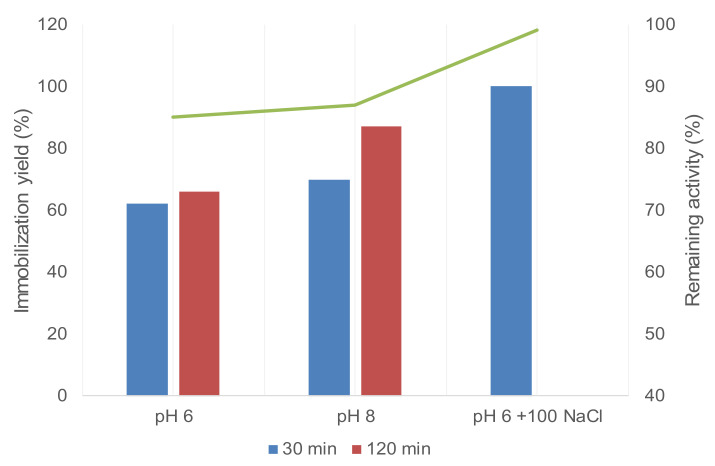
Immobilization of GTLC193_N_3_ on geranyl-SP support at different conditions.

Then, the immobilization of GTLC193_N_3_ on geranyl support was attempted at different pHs in the presence of CuI (Figure 6).

The best results were obtained at pH 8 after 2 h incubation with 87% immobilization, whereas at pH 6, the value was around 70% (Figure 6). In both cases, the lipase-retained activity on the solid was higher than 80%. Surprisingly, a faster immobilization procedure was observed at pH 6 in the presence of NaCl, where complete immobilization was observed after 30 min incubation, with almost full activity conserved (Figure 6).

This effect is in concordance with the previous results in CuAAC, which demonstrate that this click reaction is favored by the presence of NaCl in the medium [39].

## 3. Materials and Methods

### 3.1. General

Lipases from *Thermomyces lanuginosus* (TLL) (Lipozyme TL100L), *Candida antarctica* (fraction B) (CAL-B) (Lipozyme CALBL), Lecitase ultra (LECI), and *Rhizomucor miehei* (RML) (Palatase 20000L) and β-galactosidase Lactozym were kindly donated by Novozymes (Bagsværd, Denmark). Lipase from *Pseudomonas fluorescens* (PFL) (Sigma-Aldrich, cat. no 534730, St. Louis, MI, USA), Lipase from *Candida rugosa* (CRL) (Sigma-Aldrich, cat. no. L1754), betagalactosidase from *E. coli*, geranyl-amine, *N*-hydroxysuccinimide (NHS), carboxymethylcelullose (CM), 1-*O*-(4-nitrophenyl)-β-d-galactopyranoside (pNP-Gal), diisopropylcarbodiimide (DIC), trimethylamine, p-nitrophenylbutyrate (pNPB), selenometheonine, 4-azide-l-homoalanine, and l-DOPA were from Sigma-Aldrich (Saint Louis, MO, USA). Tyrosine from *A. bisporus* was produced as reported [40]. 1-Ethyl-3-(3-dimethylaminopropyl) carbodiimide (EDC) was acquired from Tokyo Kasei (Tokyo, Japan). SB-IDA405 Sepabeads (SP) resin was from Resindion srl (Binasco, Italy). The scanning electron microscopy (SEM) imaging was performed on a TM-1000 Hitachi (Tokio, Japan) microscope. The X-ray diffraction (XRD) pattern was obtained using a Texture Analysis Diffractometer D8 Advance Bruker (Billerica, MA, USA) with Cu Kα radiation. The spectrophotometric analyses were run on a V-730 spectrophotometer JASCO (Tokio, Japan).

### 3.2. Preparation of the Geranyl-Functionalized Support

One gram of CM (1 mmol/g) or IDA-Sepabeads (SP) was suspended in 10 mL of distilled water adjusted at pH 5. Then, 100 μL of geranyl-amine (82 mg, 0.53 mmol) were added to 100 μL of pure ACN (this will correspond to 50% of the modified groups). This latter solution was added drop by drop to the support suspension. Afterwards, a solid 1.9 g of EDC (10 eq) were added, and the mixture was incubated overnight. Finally, the solid was filtered by vacuum, washed with abundant distilled water (3 × 200 mL), filtered, and store at 4 °C. Also for SP, one-gram support was suspended in 10 ml of dioxane and 5 equiv of geranyl-amine, 10 equiv of DIC and 10 equiv of trimethylamine were added (full modification). After 2 h incubation, the solid was filtered by vacuum, washed with abundant distilled water (3 × 200 mL), filtered, and store at 4 °C.

### 3.3. Enzymatic Activity Assays

#### 3.3.1. Lipase Activity Assay

The standard activity assay was performed by measuring the increase in absorbance at 348 nm (isobastic point of p-nitrophenol) produced by the releasing of p-nitrophenol in the hydrolysis of 0.4 mM p-nitrophenyl butyrate in 25 mM sodium phosphate at pH 7 and 25 °C, using a thermostatized spectrophotomer with magnetic stirring. To initialize the reaction, 0.1 mL of lipase solution or suspension was added to 2.5 mL of substrate solution. An international unit of pNPB activity is defined as the amount of enzyme necessary to hydrolyze 1 µmol of pNPB/min (IU) under the conditions described above.

#### 3.3.2. Tyrosinase Activity Assay

Tyrosinase activity was tested in the presence of 2 mL of 1 mM L-DOPA in 0.1 M phosphate buffer pH 7 at room temperature using a V-730 spectrophotometer (Jasco, Tokio, Japan), measuring the increase in absorbance of aminochromes at 475 nm caused by 40 µL of enzyme solution, and taking the initial speed between 10 and 70 s of the reaction. One unit of enzyme activity (U) was defined as the amount of enzyme that causes a 0.001 increase in absorbance/min at 25 °C.

#### 3.3.3. Glycosidase Activity Assay

The standard activity assay was performed by measuring the increase in absorbance at 405 nm (isobastic point of o-nitrophenol) produced by the releasing of o-nitrophenol in the hydrolysis of 13 mM o-nitrophenyl-β-d-galactopyranoside (oNPG) in 50 mM sodium phosphate at pH 7 and 25 °C, using a thermostatized spectrophotomer with magnetic stirring. To initialize the reaction, 0.1 mL of β-Galactosidase from *E. coli* solution or suspension was added to 2 mL of substrate solution. An international unit of oNPG activity is defined as the amount of enzyme necessary to hydrolyze 1 µmol of oNPG/min (IU) under the conditions described above.

### 3.4. Immobilization of Enzymes on Geranyl-Support by Hydrophobic Interactions

One gram of geranyl–Sepabeads/CM support was added to 10 mL of protein solution (0.7–1.2 mg lipase/mL or 0.25 mg/mL tyrosinase in 10 mM sodium phosphate pH 7) or 6 mL of (0.35 mg/mL for b-gal, 0.27 mg/mL for 0440, 0.45 mg/mL for 3525) at 25 °C and under very mild stirring (see Table 1). The activities of the supernatants and immobilization suspensions were periodically checked by the method described above or by Bradford assay. After 2 h, the immobilized enzyme was filtered, washed, and vacuum dried for its later use.

### 3.5. Covalent Immobilization of SH-, Se, or N_3-_Containing Enzymes on Geranyl Support

One gram of geranyl Sepabeads support was added to 10 mL of modified GTL variants (GTLC193, GTLC334, GTLC193_SeH, GTLC193_N_3_) or RML_SH or BGal_SH solution at 25 °C at different pHs and under very mild stirring. The activities of the supernatants and immobilization suspensions were periodically checked by the method described above. After 2 h, the immobilized enzyme was filtered, washed with abundant distilled water (2 × 200 mL), filtered, and stored at 4 °C.

## 4. Conclusions

We have developed a novel prenylated functionalized material that allows immobilizing proteins by different selective strategies at room temperature and aqueous media. An enzyme can be selectively immobilized by reversible hydrophobic interaction whereas it is able to immobilize the same enzyme previously functionalized with SH, Se, or N3 groups by selective covalent interactions simply with slight modification in the experimental conditions (mainly pH). Therefore, this strategy allows co-immobilizing enzymes on the same support (the same enzyme such as lipase, or different enzymes) in a very simple and efficient way, which represents a new alternative for the creation of novel biocatalysts for cascade processes.

## Figures and Tables

**Table 1:** Immobilization of enzymes on geranyl support at pH 7 buffer phosphate 25 mM.

Entry	Protein ^1^	Support	Mg Protein/g Support	Immobilization Yield (%) ^2^	Retained Activity (%) ^4^
1	CAL-B	Geranyl-SP	9.0	96	149
2	TLL	Geranyl-SP	9.2	92	91
3	RML	Geranyl-SP	7.2	84	182
4	ANL	Geranyl-SP	8.8	70	105
5	CRL	Geranyl-SP	8.2	81	15
6	PFL	Geranyl-SP	8.4	71	20
7	LECI	Geranyl-SP	12	95	51
8	Tyr	Geranyl-CM	3	40	nd
9	Tyr	Geranyl-SP	3	90	50
10	β-Gal/ Lac	Geranyl-SP	2.12/11	0	-
11	Lp_0440	Geranyl-CM	1.62	>95 ^3^	nd
12	Lp_3525	Geranyl-CM	2.7	66 ^3^	nd

^1^ ANL: *Aspergillus niger* lipase; RML: *Rhizomucor miehei* lipase; CALB: *Candida antarctica* B lipase; CRL: *Candida rugosa* lipase; TLL: *Thermomyces lanuginosus* lipase; LECI: Lecitase Ultra^®^ phospholipase; PFL: *Pseudomonas fluorescens* lipase; Tyr: tyrosinase from *A. bisporus*, β-Gal: betagalactosidase from *E. coli*, Lac:Lactozym (betagalactosidase from *A. niger*), Lp_0440: phosphoglucosidase from *L. plantarum* WCFS1, Lp_3525: phosphoglucosidase from *L. plantarum* WCFS1. ^2^ Immobilization yield after 3 h incubation calculated by enzymatic activity. ^3^ Immobilization yield was determined by Bradford assay and confirmed by SDS-PAGE. ^4^ Activity in the pNPB assay showed of the enzymes on the solid. This value is calculated by comparing with the initial activity value achieved by the enzyme in solution (which is 100%). Nd: no determined.

**Table 2:** Immobilization of cysteine-containing enzymes on geranyl-SP support at pH 8.

Protein ^1^	Additive	pH	Mg Protein/g Support	Immobilization Yield (%) ^4^	Retained Activity (%) ^5^
GTLC193	Triton X-100 ^2^	5	10	<10	90
GTLC193	Triton X-100 ^2^	6	10	<10	90
GTLC193	Triton X-100 ^2^	8	10	42	84
GTLC334	Triton X-100 ^2^	8	10	64	48
RML_SH	Lauryl Sucrose ^3^	8	14	64	99
B-Gal_SH	Triton X-100 ^2^	8	0.8	79	99

^1^ GTL: *GeoBacillus Thermocatenulatus* lipase. RML: *Rhizomucor miehei* lipase; β-Gal: beta galactosidase from *E. coli.* ^2^ 0.1% *(v/v)*. ^3^ 1% *(v/v)*. ^4^ Immobilization yield after 2 h incubation calculated by enzymatic activity. ^5^ Activity showed of the enzymes on the solid. This value is calculated by comparing with the initial activity value achieved by the enzyme in solution (which is 100%).

## Data Availability

Not applicable.

## Supplementary Materials

The following are available online. Figure S1: SEM images of dried CM. Figure S2: SEM images of dried geranyl functionalized-CM. Figure S3: SEM images of dried IDA-Sepabeads. Figure S4: SDS-page of the different lipases and immobilized preparations on geranyl-support. Figure S5: Lp_0440 structure incorporating a His6-tag in the C-terminus. Figure S6: GTL sequence and determination of methionines. Figure S7: (A) Three-dimensional structure of the *G. thermocatenulatus* lipase in open conformation marking superficial methionine residues (red). (B) Picture of the methionines in the protein structure location. Figure S8: SDS-PAGE of the purification of GTLC193_SeH and GTLC193_N3 variants. Table S1. Immobilization of enzymes on octyl-Sepharose at pH 7 buffer phosphate 25 mM.

## Sample Availability

Samples of the compounds used in this study are available from the authors.
