# Supplementary material for: Geranyl Functionalized Materials for Site-Specific Co-Immobilization of Proteins
Source: Molecules. 2021 May 19;26(10):3028. doi: 10.3390/molecules26103028 (PMC8161341; doi:10.3390/molecules26103028)
Supplement: Supplementary file 1 [file molecules-26-03028-s001.zip › molecules-1211850-supplementary.pdf]

Supplementary

Geranyl Functionalized Materials for Site-Specific Co-Immobilization of Proteins

Jana Brabcova [1,2], Alicia Andreu [1], David Aguilera [1], Zaida Cabrera[3], Blanca de las Rivas [2], Rosario Muñoz [2] and Jose M. Palomo [1,*]

Experimental

Site-directed mutagenesis of GTL

All site-directed mutagenesis experiments were carried out by PCR using mutagenic primers (Table S1 of the supporting information). Further digestion with endonuclease *DpnI* to eliminate template DNA was done. A double mutant was sequentially carried out for GTL. Firstly, by using plasmid pT1GTL as template, pT1GTLC65S plasmid was constructed encoding GTL protein having a C65S mutation. Subsequently, this plasmid was used to construct a plasmid encoding a double mutant GTL protein carrying C65S and C296S mutations. Finally, this latter plasmid was used as template to construct additional mutations (A193C or T343S) using different mutagenic primers (see below). The sequence of the resulting plasmids encoding mutated GTL was confirmed by DNA sequencing.

Mutant	Plasmid template	Primers[b] (5´-3´)
C65S	pT1GTL	Forward-CAACTGGGACCGGGCGagcGAAGCGTACGCCCAG<br>Reverse-CTGGGCGTACGCTTCgctCGCCCGGTCCCAGTTG
C65S C296S[a]	pT1GTLC65S [b]	Forward-GCGGTCGTAagcGCCCCGTTTC<br>Reverse-GAAACGGGGCgctTACGACCGC
A193C	pT1GTLmutCys[c]	Forward-GAAAGCGtgcGCTGTCGCC<br>Reverse-GGCGACAGCgcaCGCTTTC
S334C	pT1GTLmutCys[c]	Forward-CGGTCCAAAGCGTGGATCAtgcGATCGGATCGTGCCGTATGACGGG<br>Reverse -CCCGTCATACGGCACGATCCGATCgcaTGATCCACGCTTTGGACCG

[a] The mutant name shows the amino acid changes and its position in GTL. [b] To eliminate both Cys residues, first was eliminate Cys64 and then was used this mutated plasmid as template to eliminate the Cys residue at position 295. [c] Plasmid with GTL mutant lacking both of the two native Cys residues (Cys64 and Cys 295).

Purification of GTL variants

The crude extract from *E. coli* containing GTL variant was diluted four times with 25 mM sodium phosphate at pH 7.0 to a concentration of 5 mg/ml. Then, butyl-Sepharose was added in a 1/10 (v/v) proportion and gently stirred for three hours at 25ºC. Periodically, activity of suspensions and supernatants were measured by the *p*NPP assay. After that, adsorbed lipase preparation was abundantly washed with distilled water. GTL-S193 or GTL-S334 was desorbed from the support by suspending the immobilized enzyme in a

1/10 (w/v) ratio in 25 mM sodium phosphate buffer at pH 7.0 containing 0.5% (v/v) of Triton X-100 during 1 hour at room temperature.

Following these protocols, a quantitative immobilization of lipase activity was observed and the SDS-PAGE analysis of the adsorbed protein preparation showed a single band with a molecular weight corresponding to the native lipase. Final purification overall yield was 95%. A solution of 0.5 mg lipase purified /mL was obtained.

Expression and Purification of the Recombinant phosphoglucosidases Lp_ 0440, and Lp_3525

The *lp_0440* and *lp_3525* genes from *L. plantarum* WCFS1 were amplified by polymerase chain reaction (PCR). Oligonucleotides 1666 (5′-AACTTTAAGAAGGAGATATACATATGACGATTAAAGGACGAGCGTTTC) and 1667 (5′-GCTATTAATGATGATGATGATGATGCTCAATTTCGGCACCATTTGTCGC) were used to amplify *lp_0440,* and finally, oligonucleotides 427 (5′-CATCATGGTGACGATGACGATAAGATGTCAGAGTTCCCAGAA) and 428 (5′-AAGCTTAGTTAGCTATTATGCGTACTATTTCTTTGTCAGCCCATTATGC) to amplify the *lp_3525* gene. Genbank accession numbers of these enzymes are WP_003642889.1 (Lp_3525) and WP_003642002.1 (Lp_0440). Advantage HD DNA polymerase (TaKaRa Biotechnology, Japan) was used for PCR amplifications. The purified PCR products were inserted into the pURI3 (*lp*_3525 gene) or pURI3-Cter (*lp*_0440 gene) vectors using a restriction enzyme- and ligation-free cloning strategy [26]. These vectors produce recombinant proteins having a six-histidine affinity tag at their N-(Lp_3525) or C-terminal (Lp_0440) ends. *Escherichia coli* DH10B cells were transformed and the recombinant plasmids obtained (pURI3-Cter-*lp*_0440 and pURI3-*lp*_3525) were isolated and verified by DNA sequencing, and then used to transform *E. coli* BL21(DE3) cells for expression. *E. coli* cells were grown in Luria Bertani broth (LB) medium containing ampicillin (100 μg/mL), until they reached an optical density at 600 nm of 0.4 and induced by adding isopropyl β-D-1-thiogalactopyranoside (IPTG) (0.4 mM final concentration). After induction, the cells were grown at 22 °C during 20 h. The induced cells were harvested by centrifugation (8000 g for 15 min at 4 °C), resuspended in phosphate buffer (50 mM, pH 7) containing 300 mM NaCl, and disrupted by French press passages (three times at 1100 psi). Insoluble fraction of the lysates was removed by centrifugation at 47000 g for 30 min at 4 °C. The supernatant was filtered through a 0.2-μm pore-size filter and then loaded onto a Talon Superflow resin (Clontech, Mountain View, CA, USA) equilibrated in phosphate buffer (50 mM, pH 7) containing 300 mM NaCl and 10 mM imidazole. The bound enzymes were eluted using 150 mM imidazole in the same buffer. The purity of the enzymes was determined by sodium dodecyl sulfate-polyacrylamide gel electrophoresis (SDS-PAGE) in tris-glycine buffer. Fractions containing the His_6-tagged protein were pooled (fractions were dialyzed against 50 mM sodium phosphate buffer, 300 mM NaCl, pH 7 at 4 °C.

Chemically preparation of cysteine-containing enzymes

The chemical incorporation of HS groups to the enzyme was performed as follow. The chemical modification is carried out in four steps: i) Immobilization on octyl-Sepharose: 0.25 g octyl-Sepharose support were incubated with 5 mL of an enzymatic solution (0.1 mg protein/mL in sodium phosphate buffer pH 7.0 10 mM). After 12 hours under gentle agitation, the suspension (immobilized lipase) was filtered, washed and stored for its later use. ii) Modification by SPDP: 0.25 g immobilized preparation (octyl-enzyme) were incubated with 5ml of phosphate buffer 500 mM pH 7.3 (for β-Gal from *E. coli*) or 5 ml of phosphate buffer 10 mM pH 7.3 (for RML) with 0.5 mg SPDP=N-succinidyl-3-(2 pyridyldithio) propionate (dissolved in 250 μL of acetonitrile 100% (v/v)). After 2 hours under gentle agitation, the suspension (modified immobilized lipase) was filtered and abundantly washed with 500 mM phosphate buffer (pH 7.3). iii) Incubation in presence of DTT: To 250mg modified immobilized enzyme (step 2) it was added 2 ml of 6.5 mM DTT in phosphate buffer 500 mM pH 7.3 for β -gal and 2 ml of 50 mM DTT in phosphate

buffer 10 mM pH 8 for RML. After 30 min under gentle agitation, the suspension (modified immobilized lipase) was filtered and abundantly washed with 500 mM phosphate buffer (pH 7.3). iv) Desorption of modified enzyme from octyl support: The modified β-gal enzyme was released from the octyl-support by incubation in 0.1% Triton X-100 in 10 mM phosphate buffer (pH 7.0) while RML was released with 1% lauryl sucrose in 25 mM phosphate buffer (pH 7.0). After 0.5 hours under gentle agitation, the suspension was filtered and the supernatant stored for its later use.

Seleno-L-methionine/4-azide-L-homoalanine hydrochloride protein expression, extraction and purification

E. coli BL21 (DE3) cells transformed with pT1GTLmutCys-A193C were incubated in LB media at 30°C during 20h and then were inoculated into 250mL of culture medium modified M9 (22.5 mL M9 media (10x), 202.5 mL sterile distilled water, MgSO4 (1M) 250µl, D-glucose (4% w/v) 25ml, Thiamine (0.5% w/v) 25µl, FeSO4 (4.2 g/l) 250µl, Ampicilin (100mg/ml) 250 µl). The cells were grown at 30°C until they reach a OD600 of 0.4 . Then were add 2ml amino acid mixture (L-lys, L-Phe, L-Thr, L-Ile, L-Val) and 12.5mg seleno-L-methionine or 12.5mg 4-Azido-L-homoalanine hydrochloride and the cells were induced by raising the temperature to 42°C for 20h.

The induced cells were harvested by centrifugation (8000g for 15min at 4 °C), re-suspended in phosphate buffer (50 mM, pH 7) containing 300 mM NaCl, and disrupted by French Press passages (three times at 1100 psi). Insoluble fraction of the lysates was removed by centrifugation at 47000g for 30 min at 4 °C. The supernatant was filtered through a 0.2 µm pore-size filter.

This supernatant obtained was offered to 1 gram of octyl-Sepharose. Later, supernatants after octyl support were offered to butyl-Sepharose (600mg).

Desorption of modified enzyme from butyl support: 300 mg of butyl_SeMet or butyl_HA was incubated in 3 ml 0.5% lauryl sucrose for 30 min at 25ºC. Subsequently, supernatants were dialyzed overnight to obtain the new GTL193_SeH and GTL193_N_3.

SDS-PAGE electrophoresis

The monitoring of immobilization process and purity of the different mutants was checked on 12% (w/v) SDS-PAGE gels electrophoresis stained with Coomasie blue. 50 mg of immobilized support were suspended in 70 µl protein loading buffer (PLB, pH 6.8 containing 0,125 mM Tris-base, bromophenol blue 10% v/v 2-mercaptoethanol, 40% glycerol, 4% SDS) or 50 µl enzyme solution (1:1) with PLB. The samples were boiled for 5 min and supernatants were used for SDS PAGE.

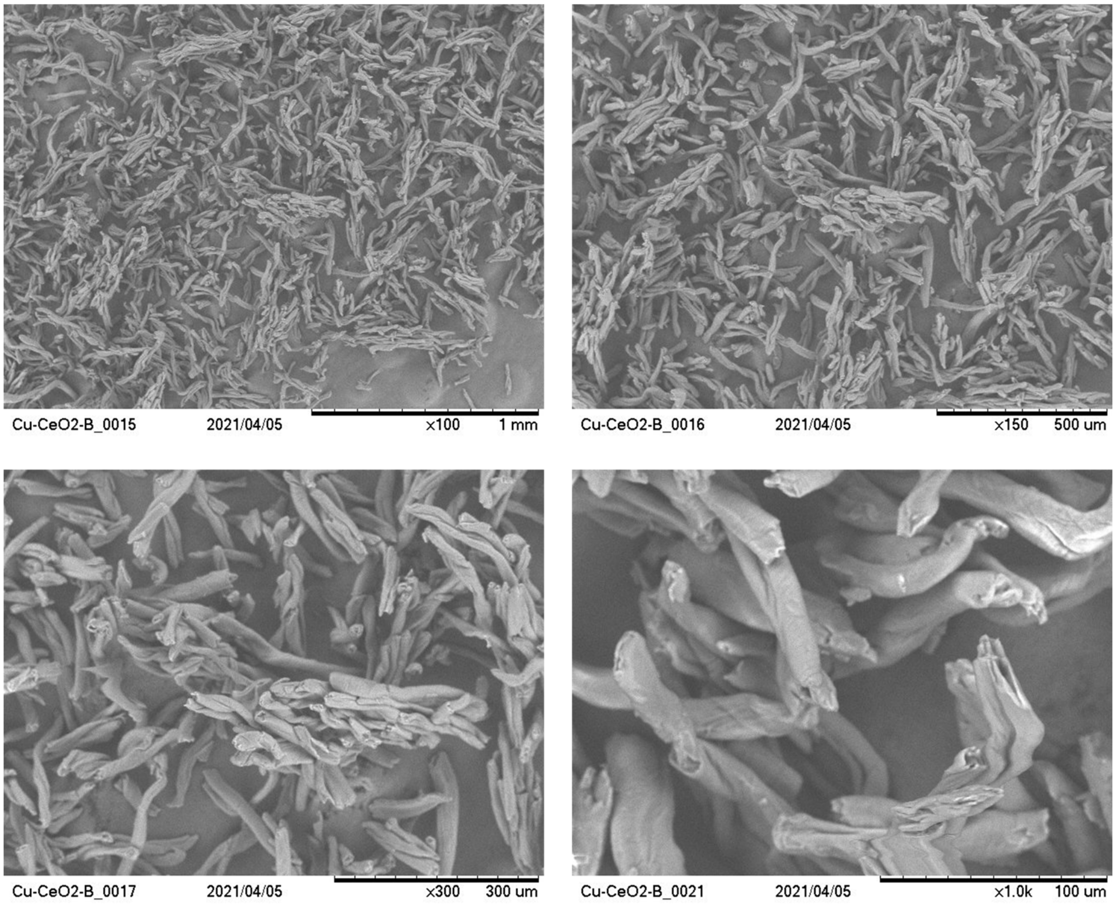

Figure S1. SEM images of dried CM.

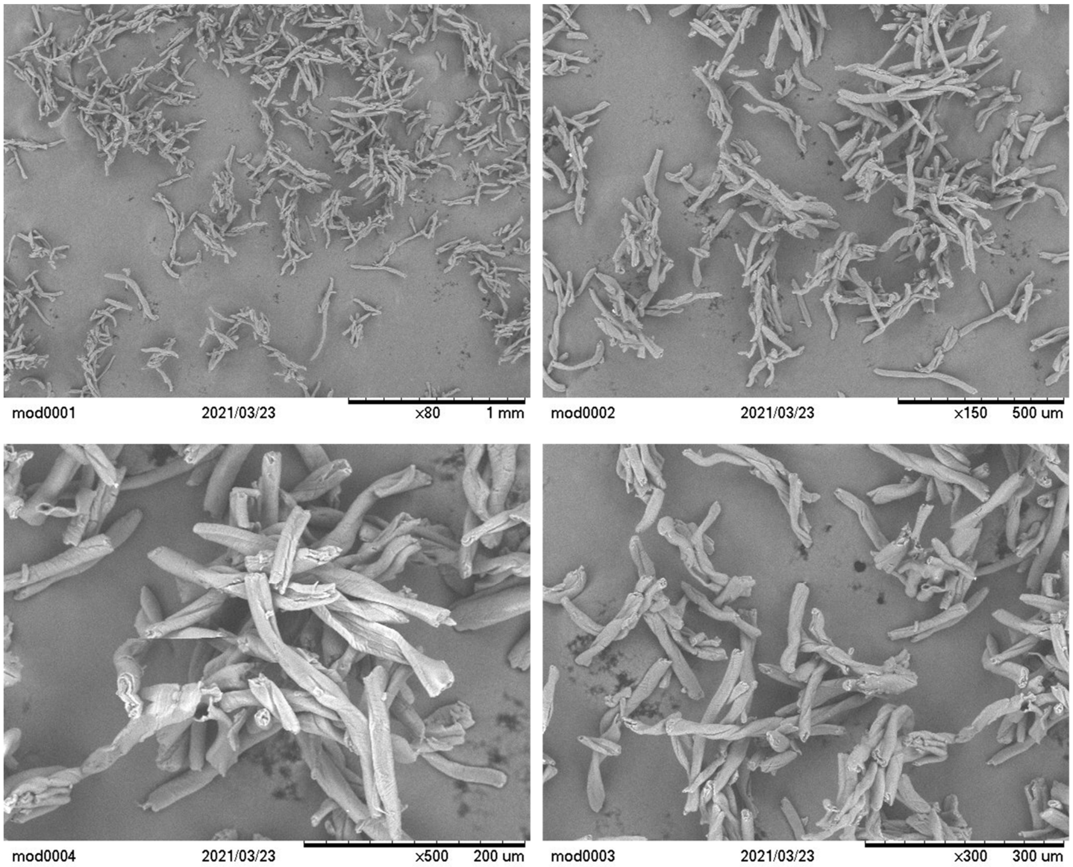

Figure S2. SEM images of dried geranyl functionalized-CM.

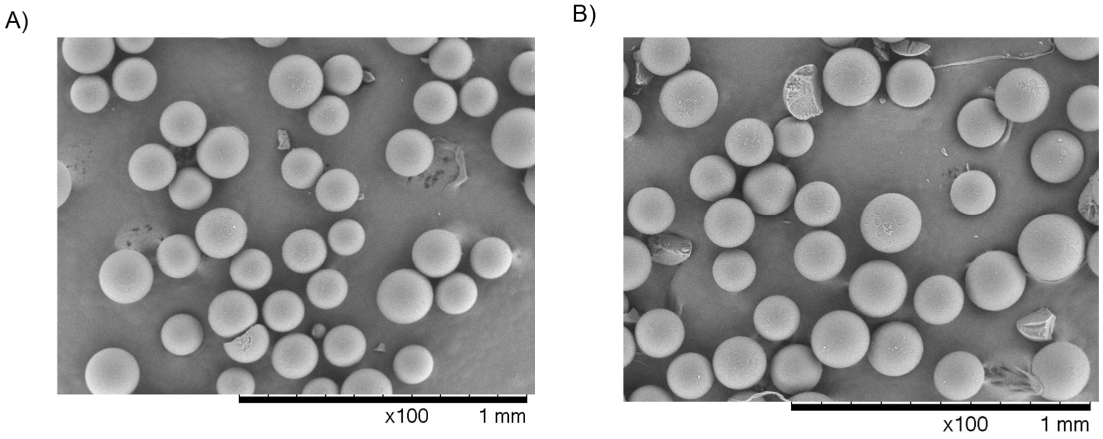

Figure S3. SEM images of dried IDA-Sepabeads. A) unfunctionalized. B) geranyl functionalized support.

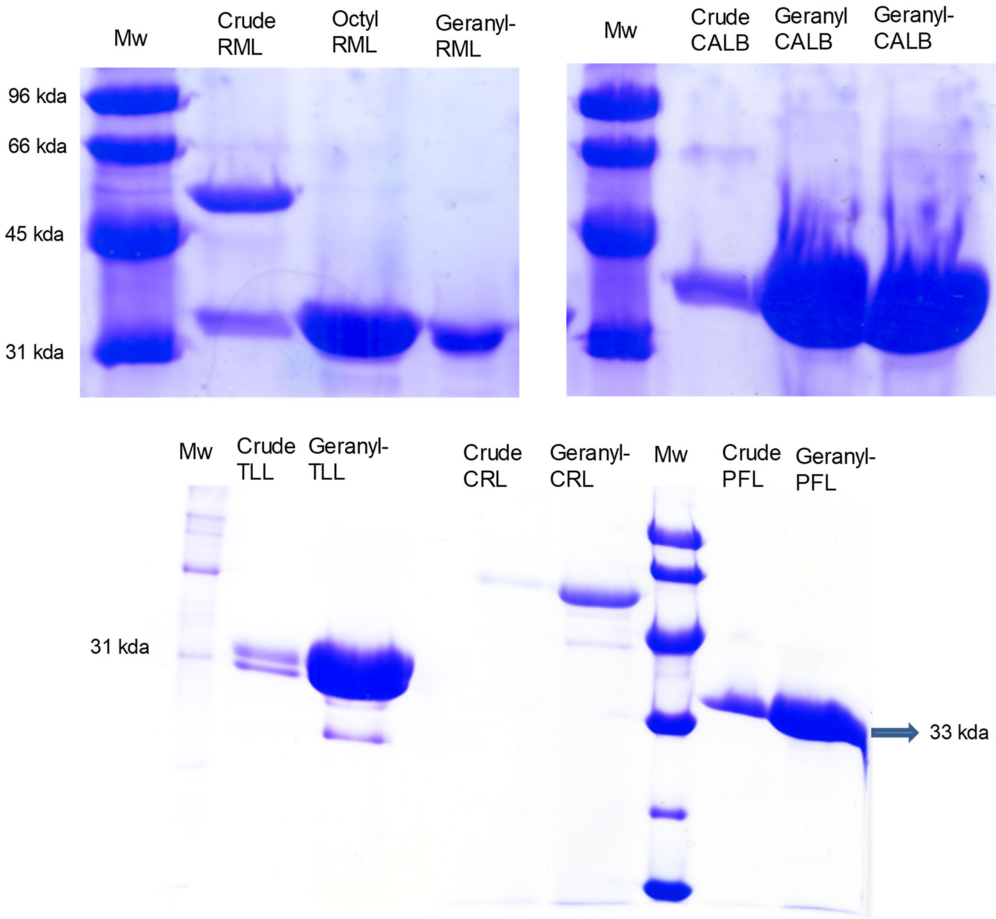

Figure S4. SDS-page of the different lipases and immobilized preparations on geranyl-support.

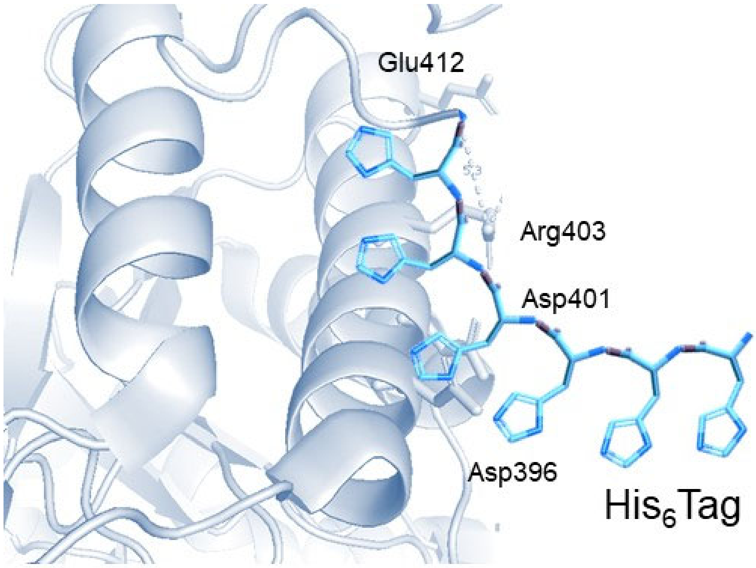

Figure S5. Lp_0440 structure incorporating a His6-tag in the C-terminus. The protein structure was obtained from the Protein Data Bank (PDB code: 3qom) and the pictures were created using Pymol v. 0.99.

GTL sequence

MASPRANDAPIVLLHGFTGWG**REEMLGFK**YWGGVRGDIEQWLNDNGYRTYTLAVGPLSSNWDRACEAYAQ
LVGGTVDYGAAHAAKHGHARFGRTYPGLLPELKRGGRVHIIAHSQGGQ**TARMLVSL**LENGSQEEREYAKA
HNVSLSPLFEGGHHFVLSVTTIATPHDGTT**LVNMVD**FTDRFFDLQKAVLKAAAVASNVPYTSQVYDFKLD
QWGLRRQPGESFDHYFERLKRSPVWTSTDTARYDLSIPGAEKLNQWVQASPNTYYLSFSTERTHRGALTG
NYY**PELGMNAF**SAVVCAPFLGSYRNEALGIDDRWLENDGIVN**TVSMNGP**KRGSSDRIVPYDGTLKKGV**WN**
DMGTYNVDHLEVIGVDPNPSFDIRAFYLRLAEQLASLRP

Exposed methionines

Met1: MASPR

Met25: REEMLGFK

Met122 : TARMLVS

Met174: LVNMVDF

Met288: PELGMNAF

Met352: WNDMGT

No exposed methionines

Met326: TVSMNGP

Figure S6. GTL sequence and determination of methionines (in red).

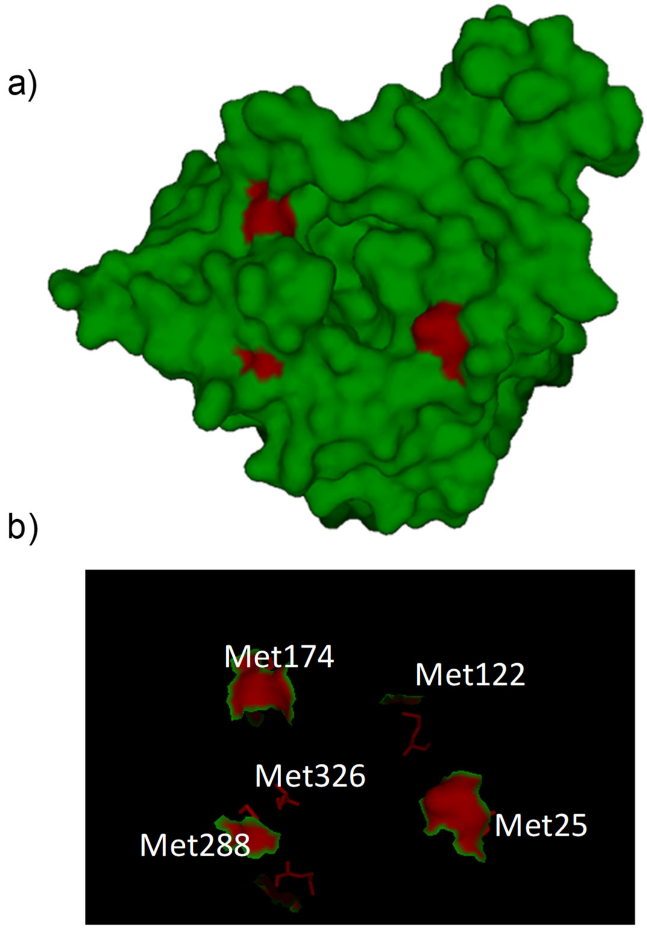

Figure S7. A) Three-dimensional structure of the *G. thermocatenulatus* lipase in open conformation marking superficial methionine residues (red). B) Picture of the methionine residues in the protein structure location. The protein structure was obtained from the Protein Data Bank (pdb code: 2W22) and the picture was created using Pymol v. 0.99.

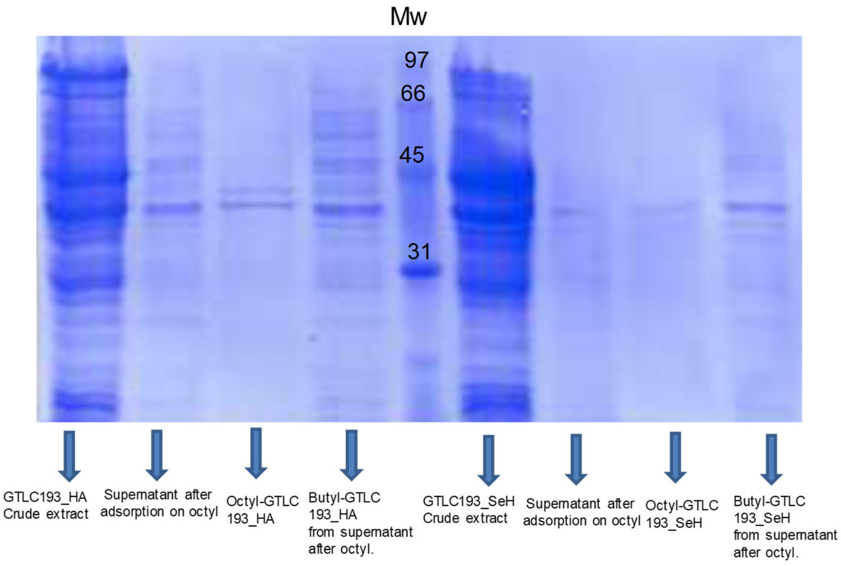

Figure S8. SDS-PAGE of the purification of GTLC193_SeH and GTLC193_N_3 variants.

Table S1. Immobilization of enzymes on octyl-Sepharose at pH 7 buffer phosphate 25 mM.

Protein[1]	Mg protein/g support	Immobilization Yield (%)[2]	Retained activity (%)[3]
CAL-B	8.5	60	80
TLL	5.5	99	99
RML	0.6	96	115
ANL	0.8	36	300
CRL	13	96	59
PFL	14	44	99
LECI	4.4	62	167
Tyr	0.4	30	nd
B-Gal	0.8	43	107
Lactozym	11	10	107

[1] ANL: *Aspergillus niger* lipase; RML: *Rhizomucor miehei* lipase; CALB: *Candida antarctica* B lipase ; CRL: *Candida rugosa* lipase; TLL: *Thermomyces lanuginosus* Lipase; LECI: Lecitase Ultra® phospholipase; PFL: *Pseudomonas fluorescens* lipase; Tyr: tyrosinase from *A. bisporus*, B-Gal: betagalactosidase from *E coli,* Lactozym: betagalactosidase from *A. niger*. [2] Immobilization yield calculated by enzymatic activity. [3]Activity showed of the enzymes on the solid. This is compared with the value achieved by the enzyme in solution (which is 100%). Nd: no determined.
